# ELiAH: the atlas of E3 ligases in human tissues for targeted protein degradation with reduced off-target effect

**DOI:** 10.1093/database/baae111

**Published:** 2024-10-12

**Authors:** Hyojung Paik, Chunryong Oh, Sajid Hussain, Sangjae Seo, Soon Woo Park, Tae Lyun Ko, Ari Lee

**Affiliations:** Center for Supercomputing Application, Korea Institute of Science and Technology Information (KISTI), 245 Daehak-ro, Daejeon 34141, South Korea; Center for Biomedical Computing, Korea Institute of Science and Technology Information (KISTI), 245 Daehak-ro, Daejeon 34141, South Korea; Department of Data and HPC Science, University of Science and Technology (UST), 245 Daehak-ro, Daejeon 34141, South Korea; Center for Supercomputing Application, Korea Institute of Science and Technology Information (KISTI), 245 Daehak-ro, Daejeon 34141, South Korea; Center for Biomedical Computing, Korea Institute of Science and Technology Information (KISTI), 245 Daehak-ro, Daejeon 34141, South Korea; Department of Data and HPC Science, University of Science and Technology (UST), 245 Daehak-ro, Daejeon 34141, South Korea; Department of Applied AI, University of Science and Technology (UST), 245 Daehak-ro, Daejeon 34141, South Korea; Center for Supercomputing Application, Korea Institute of Science and Technology Information (KISTI), 245 Daehak-ro, Daejeon 34141, South Korea; Center for Nanotubes and Nanostructured Composites, Sungkyunkwan University, 2066 Seobu-ro, Suwon 16419, South Korea; Center for Biomedical Computing, Korea Institute of Science and Technology Information (KISTI), 245 Daehak-ro, Daejeon 34141, South Korea; Department of Data and HPC Science, University of Science and Technology (UST), 245 Daehak-ro, Daejeon 34141, South Korea; Center for Supercomputing Application, Korea Institute of Science and Technology Information (KISTI), 245 Daehak-ro, Daejeon 34141, South Korea; Center for Biomedical Computing, Korea Institute of Science and Technology Information (KISTI), 245 Daehak-ro, Daejeon 34141, South Korea

## Abstract

The development of therapeutic agents has mainly focused on designing small molecules to modulate target proteins or genes which are conventionally druggable. Therefore, targeted protein degradation (TPD) for undruggable cases has emerged as promising pharmaceutical approach. TPD, often referred PROTACs (PROteolysis TArgeting Chimeras), uses a linker to degrade target proteins by hijacking the ubiquitination system. Therefore, unravel the relationship including reversal and co-expression between E3 ligands and other possible target genes in various human tissues is essential to mitigate off-target effects of TPD. Here, we developed the atlas of E3 ligases in human tissues (ELiAH), to prioritize E3 ligase–target gene pairs for TPD. Leveraging over 2900 of RNA-seq profiles consisting of 11 human tissues from the GTEx (genotype-tissue expression) consortium, users of ELiAH can identify tissue-specific genes and E3 ligases (FDR *P*-value of Mann–Whitney test < .05). ELiAH unravels 933 830 relationships consisting of 614 E3 ligases and 20 924 of expressed genes considering degree of tissue specificity, which are indispensable for ubiquitination based TPD development. In addition, docking properties of those relationships are also modeled using RosettaDock. Therefore, ELiAH presents comprehensive repertoire of E3 ligases for ubiquitination-based TPD drug development avoiding off-target effects.

**Database URL**: https://eliahdb.org

## Introduction

Adequate and precise degradation of disease-associated genes or proteins are essential for the success of a development a medicinal chemical. However, conventional drug discovery mainly focuses on a regulation of a limited number of target protein showing druggable features, such as a specific surface marker in a cell. Targeted protein degradation (TPD) including PROteolysis TArgeting Chimeras (PROTACs) offers several potential advantages over traditional small molecule inhibitors, including: (i) TPD can target proteins considered “undruggable” by traditional methods, including proteins lacking suitable binding pockets (ii) TPD technology can achieve selective degradation of target proteins by high-jacking E3 ligase derived ubiquitination system in a cell. Although there are diverse modes of action in TPD [1], PROTACs use E3 ligase-triggered ubiquitination. This unique mechanism of PROTAC mediates the ubiquitin-proteasome system inherent in the human body as a binder then degrades a linked protein by forming a ternary complex [2–4]. Since the first development of PROTACs by Sakamoto *et al*. [4], it has been successfully applied for TPD [5] leading clinical trials (ARV-110, ARV-471).

Knowledge-based approaches have been utilized to establish a reference repertoire of PROTACs. For example, Weng *et al*. developed PROTAC-DB by conducting extensive literature analysis, which predominantly explores the chemical properties of PROTACs, covering aspects such as their chemical structures, biological activities, and physicochemical properties [6]. Likewise, UbiNet scrutinized reliable interactions between E3 ligases and their substrates based on the literature [7]. Even though these previous attempts provided a comprehensive understanding of existing PROTACs, the information to reduce non-specific ubiquitination of target protein, such as tissue specificity of E3 ligases and target genes, is rarely presented.

The bifunctional nature of PROTACs refers to their ability to simultaneously engage two different proteins within a cell: the target protein of interest and an E3 ubiquitin ligase enzyme. This dual engagement is a key feature of PROTACs and is essential for their mechanism of action in inducing TPD. Therefore, for selective degradation of protein of interest, the tissue expression profiles of E3 ligases are a central premise to develop an effective degradation of protein via PROTACs [8]. Our developed web database, E3 Ligase Atlas of Human (ELiAH), provides a comprehensive tissue atlas of E3 ligases to facilitate the advanced selection of PROTACs for development. To the best of our knowledge, focusing on tissue-specific characteristics, this is the first online database for TPD development that directly utilizes raw data of high-throughput expression profiles including FASTQ collected from over 2000 human tissues in GTEx [9]. Since we introduced an independent analysis pipeline comparing GTEx and a previous attempt of E3 Ligase analysis (https://hanlaboratory.com/E3Atlas/), ELiAH supports a cross-reference for the use of GTEx for TPD development. The tissue specificity of those E3 ligases and associated genes including target genes are also introduced with a user-friendly web interface.

In ELiAH (http://eliahdb.org), through mutual information [10] analysis, we identified a total of 933 830 relationships between E3 ligases and expressed genes across tissues, potentially enabling the development of tissue-specific PROTACs. Additionally, ELiAH provides diverse information including cross-referencing of the identified relationships in the protein–protein network drawn from STRING [11]. Using RosettaDock, ELiAH introduce binding property between E3 ligase–substrate pairs. All data from ELiAH, including tissue specificity, relationship between E3 ligases and genes, and docking properties, are available for download as Comma-Separated Value files.

## Materials and methods

### Data preparation and analysis

We opted to utilize raw RNA-seq data from dbGAP instead of using preprocessed data from the GTEx web repository [9]. This approach allowed us to maintain control over data quality and select non-tumor samples based on aligned phenotype data of each individual. We filtered 655 out of 2947 samples from the GTEx based on the estimated alignment coverage of HISAT2 [12] (>80% of alignment). Salmon [13] and DESeq2 [14] were used for sample-wide normalization and gene expression quantification. Using R, the tissue specificity of each gene was determined based on the *P*-value of the Mann–Whitney test. With those expression profiles, the association between E3 ligases and genes was addressed using mutual information [10]. Flexible docking analyses were conducted through RosettaDock to examine the molecular interactions between E3 ligase and target protein [15]. The PDB files required for these analyses were acquired from the AlphaFold database, utilizing UniProt identifiers that were retrieved from the associated gene names [16]. The global docking protocol of RosettaDock was employed, wherein for each interacting pair, a set of 100 decoy models was created. Subsequently, the decoy with the minimum docking score for each pair was recorded in our database.

### Design of web database

ELiAH is hosted on an Apache web server and powered by Apache Tomcat to ensure seamless operation (https://httpd.apache.org/, https://tomcat.apache.org/). The core functionality of ELiAH is built upon the Flask web framework (https://flask.palletsprojects.com/en/3.0.x/), incorporating asynchronous networking techniques to ensure efficient handling of requests. ELiAH is deployed on a Linux server, providing user accessibility at http://eliahdb.org/. All data are stored in MySQL, a robust object-relational database (https://www.mysql.org/). The database integrates the reference information drawn from the STRING database (https://string-db.org/), by using the API of STRING, ELiAH provide protein–protein interaction network image of gene that queried by user, providing valuable insights into E3 ligase-associated gene interactions. Using D3.js, our database presents level of expression abundances (i.e. *z*-score normalized expression values) of the queried gene sets in diverse human tissues.

## Results

### Database contents and statistics

As outlined in the “Materials and methods” section, ELiAH utilizes high-throughput data obtained from bulk RNA-seq analysis of over 2000 human tissues. To filter out samples associated with non-normal state including bone marrow cell-derived leukemia cell line, we performed comprehensive analysis of all sample data from the FASTQ level instead of relying on preprocessed data from GTEx. This also may allow us further integrative analysis including expansion of ELiAH by comparing patient’s derived RNA-seq data. Out of 9000 samples of GTEx (ver. 2), we prioritized 10 tissues for further analysis. Those 10 tissues showed over 100 samples per each tissue. We acknowledge brain tissues were excluded considering the heterogeneity of aging impact. Finally, 10 tissues with 2947 samples used for further analysis (Table 1).

**Table 1.** ELiAH data content and statistics

Data	Frequency
Total number of samples in GTEx (ver. 2.0)	9247
Number of selected samples (>100 samples for each tissue group)	2947
Number of analyzed samples (mean of alignment 93.4%)^a^	2292
Gastrointestinal tissue (stomach, colon—transverse, sigmoid)	544
Cardiac tissue (left ventricle, atrial appendage)	411
Muscle—skeletal	366
Whole blood	291
Thyroid	272
Lung	225
Pancreas	183
Number of E3 ligases	614
Tissue-specific E3 ligases (FDR of *P* < .05)^b^	13
Number of associated E3 ligase-target gene (FDR of *P* < .05)^c^	933 830
Gastrointestinal tissue (stomach, colon—transverse, sigmoid)	195 708
Cardiac tissue (left ventricle, atrial appendage)	186 797
Whole blood	166 225
Muscle—skeletal	199 792
Thyroid	248 664
Pancreas	176 833
Lung	221 630

a Samples were filtered out using the overall alignment rate of Hisat2 (<80%).

b Tissue specificity of a gene was determined by Mann–Whitney *U*-test for transcriptional expressions of each gene across tissues.

c The *P*-value of mutual information score was calculated in 100 bootstrap consolidation for indicating significance of each edge (gene pair between an E3 ligase and a target substrates) using ARACNe-AP. *P*-values were correacted by Bonferroni adjustment.

Out of 614 of E3 ligases, we evaluated tissue specificity using Mann–Whitney test (FDR of *P* < .05). Thirteen of E3 ligases are presented as tissue-specific E3 ligases. For example, ZNRF3, a known candidate of PROTAC for colon cancer [17], has been prioritized as gastrointestinal-specific E3 ligases.

### Exploration of tissue specificity of E3 ligases and associated genes

Based on the analysis of bulk RNA-seq analysis, we evaluated tissue-specific genes using statistical approach (*P*-value of Mann–Whitney test). Then, the associations between E3 ligase and target genes were identified based on mutual information of ARACNe-AP [10]. The “association” in our study indicates the “dependency” between E3 and other non-E3 genes based on the mutual information scores of the ARACNe-AP algorithm. The statistical significance of those associations is determined by the *P*-value of mutual information score. Therefore, the association covers co-expression and reverse patterns regardless of linear or nonlinear fashion.

**Figure 1.**
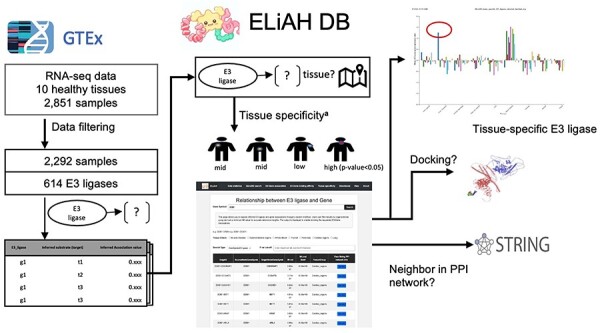
ELiAH pipeline overview.

^a^Tissue specificity was assessed by comparing the expression of E3 ligase genes across six tissues. The one-tailed Mann–Whitney U-test confirmed genes specific to each tissue with significantly higher expression compared to others. We applied the Benjamini–Hochberg test with an FDR-adjusted P-value threshold of <.05 to mitigate false positives.

Figure 1 depicts the analysis pipeline and the expected user experience. In ELiAH, users explore expression profiles of E3 ligases and human genes regarding tissue specificity. While Liu *et al*. quantified the overall abundance of E3 ligase expression using preprocessed dataset of GTEx [18], the tissue specificity of E3 ligases and their associated target genes remains to be determined. Moving forward, users of ELiAH will be able to explore the degree of tissue specificity for their queried E3 ligases and associated target genes. We have identified over 20 000 target genes associated with E3 ligases. For example, NSD2, a known oncogenic gene in colon cancer [19], has been identified as a colon-specific E3 ligase (*P*-value of Mann–Whitney *U*-test = 8.96E-80, Fig. 1). Conversely, users can deprioritize non-specific E3 ligases, such as TRIM 60 which showed continuous expression for all tissues meaning non-specific degradation of protein of interest offering off-target effect. Supplementary Fig. S1 presents more examples that show the capacity of ELiAH including a pancreas-specific gene KEAP1 and non-tissue specific E3 ligase TRIM 49. All of those explorations are presented in our user-friendly web resource, ELiAH.

To interrogate the reliability of those identified associations between E3 ligases and genes, we examined the shortest distance of each gene pair the protein–protein interaction (PPI) network via the STRING. The gene pairs with direct interactions presenting distance 1 in the PPI indicate strong association in general. In Supplementary Fig. S2, the distances between the identified associated genes and E3 ligases presented negative correlation with the mutual information value of those, meaning strong mutual information are associated more direct interaction in PPIs (*P*-value 8.60-e03). In the same way, the identified E3–gene associations showed unfavorable association with the shortest distances in the PPIs supporting the previous attempt about non-tissue-specific interactions in gastrointestinal system [20]. Therefore, the identified E3 ligases and gene associations are reliable.

### Docking analysis between E3 ligases and associated genes

Out of 933 830 of identified pairs of associations between 614 of E3 ligases and all human genes, flexible docking analyses were conducted using RosettaDock. This analysis aimed to investigate the molecular interactions at the protein–protein interface between E3 ligases and their target proteins. Docking calculations successfully generated meaningful data for 24 144 out of the 933 830 pairs. The remaining pairs either failed during the calculation or resulted in uninterpretable values. Among the 24 144 successfully analyzed pairs, 392 pairs exhibited a Rosetta Interface Score (Isc) below −30. A lower Isc generally indicates a more favorable interaction between the E3 ligase and the target protein, suggesting these pairs represent highly favorable candidates for ubiquitination. To enhance the efficiency of our analyses, we utilized the Nurion supercomputer to rapidly generate 25 decoy models for each pair of interacting E3 ligase and target protein, utilizing RosettaDock’s global docking protocol. For each pair, the decoy that achieved a successful docking score calculation was selected and recorded in our database, thereby facilitating a thorough examination of the potential binding efficiencies and the dynamics of these molecular interactions.

### Usage example: data query and browsing in ELiAH

**Figure 2.**
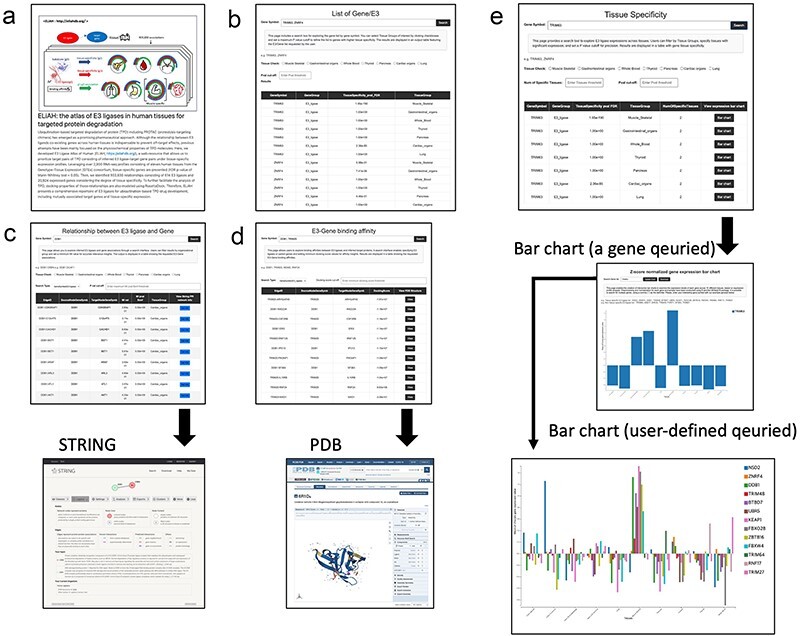
Data browsing and user experience in ELiAH.

The query and browsing results, incorporating the tissue specificity of E3 ligases determined by user-defined cut-off values, are depicted in Fig. 2. Here, we provide more detailed examples of search function of ELiAH and other supported visualization features. From the first page of ELiAH (Fig. 2a), users can explore tissue specificity not only for known E3 ligases but also for all human genes (Fig. 2b). As we described earlier, ELiAH also presents 933 830 associations between E3 ligases within each tissue. In summary, using ELiAH, users can determine tissue-specific expressions of E3 ligases, and significantly associated expression of genes, respectively. Therefore, users of ELiAH feasibly prioritize tissue-specific E3 ligases and target genes for TPD considering degree of off-target effect (Fig. 2c). Other visualization features are also presented as cross references in proteome-level (Fig. 2c and d).

(a) Front page of ELiAH. (b) Exploration page for E3 ligases and gene with user-defined queries. (c) Data query page for gene-to-gene association consisting of E3 ligase and target gene. The external information for the identified gene-to-gene association is presented based on protein–protein interactions in the STRING database. (d) Exploration page of binding affinity between E3 ligase and gene. Clicking the button displays external structural information in PDB. (e) Exploration example of tissue-specific gene and E3 ligase identification. By choosing the “bar chart” option, users of ELiAH can trace the overall transcriptional profiles across tissues based on the normalized z-score.

The ELiAH presents dynamic visualization of the degree of tissue-specificity based on the user-defined set of genes (Fig. 2e). The barchart of Fig. 2e allows direct set of query genes, and dynamic visualizations. For example, users can simultaneously visualize a colon-specific gene NSD2 and a lung-specific gene TRIM25. All visualized bar chart images are freely downloaded without log-in. Comprehensive usage details are available on our help page. Altogether, ELiAH maximize exploration feasibility of users for TPD development.

## Conclusions

By virtue of the unique mechanisms and potential advantages over traditional inhibitors, TPD technologies, including PROTACs, showed promising features in the pharmaceutical field. However, achieving specific degradation of the target protein has remained an unresolved technical hurdle for PROTACs. While degrader–antibody conjugation with PROTAC has emerged as an alternative technology [21], the transcriptional abundance of E3 ligases and associated genes remains essential for the development of drugs harnessing the ubiquitin-proteasome system. Fathom of the abundances of E3 ligases and genes across human tissues is still pending. Addressing this unmet inquiry, ELiAH leveraged large-scale transcriptional profiles in diverse human tissues suggesting the atlas of E3 ligases and associated gene expressions and their tissue specificity.

There are several aspects that require further refinement and improvement in subsequent releases. We note that the current version of ELiAH serves as a foundational reference for PROTAC development. However, based on the previous attempt of ours [22], organs and tissues are consisting of diverse types of cells. Thus, the heterogeneity of cell populations within human tissues, including tumor regions, needs to be addressed in further developments of ELiAH. For the identification of cell-specific E3 ligases and associated gene partners, big computational hurdle is expected in astronomical scale. Therefore, the development of parallel computing technology in medicinal pharmacology field for those analyses remained for further study.

Based on the raised limitations of ELiAH, we can deduce future directions of bioinformatic analysis, which would pave the way for TPD development. First, there are diverse methods to determine tissue-specific expression of genes. In principle, to quantify tissue-specificity of gene, normalized gene expression matrix across tissues is essential. So, to allow application of diverse method for identifying tissue-specific gene, we presented preprocessed gene expression matrix across all tissue samples in GTEx in the download tab. Second, we admit that linking proteomic evidences into our database would significantly improve the reliability of our E3 expression profiles and aid in identifying high-priority targets for TPD development in specific tissues. Although we present a general reference of proteome data including STRING and PDB, linking systematic experimental evidences in the Human Protein Atlas is still pending.

In conclusion, although the limitation of ours including lack of proteomic evidences, ELiAH contribute prioritization of key components of PROTAC (i.e. E3 ligase and target gene) with decreased off-target effect. We anticipate that ELiAH will function as a catalyst, accelerating research in the development of ubiquitination-based TPD drugs.

## Supplementary Material

baae111_Supp

## Data Availability

ELiAH is freely accessible online data available at: https://eliahdb.org/. Users can access this resource without registration or login. The RNA-seq data (phs000424.v2 dbGAP) of GTEx consortium can be shared with permission of GTEx consortium.
