# Supplementary figures and images for: ELiAH: the atlas of E3 ligases in human tissues for targeted protein degradation with reduced off-target effect
Source: Database (Oxford). 2024 Oct 12;2024:baae111. doi: 10.1093/database/baae111 (PMC11470751; doi:10.1093/database/baae111)

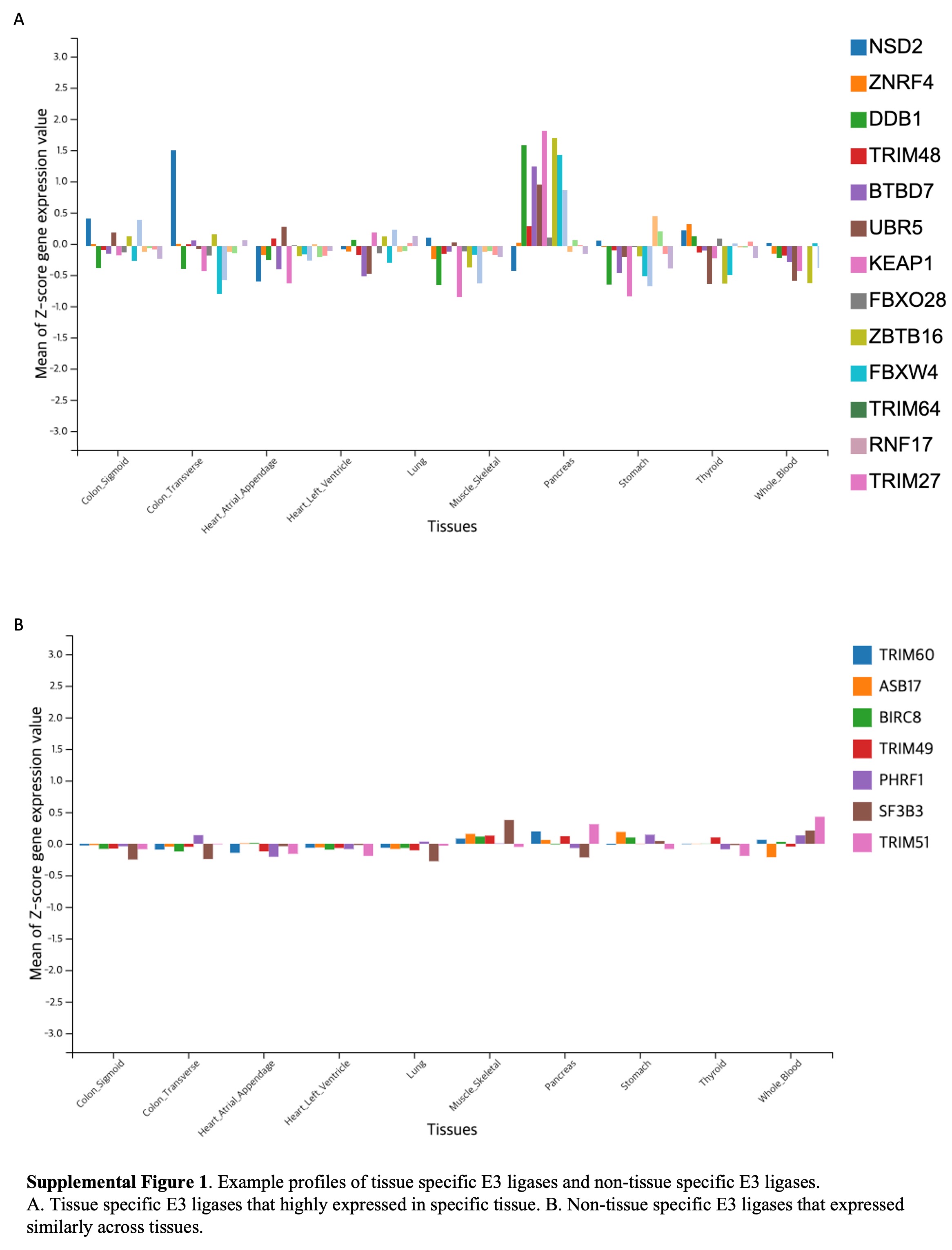

Supplement: baae111_Supp [file baae111_supp.zip › suppl_data/SFig1_original.jpg]

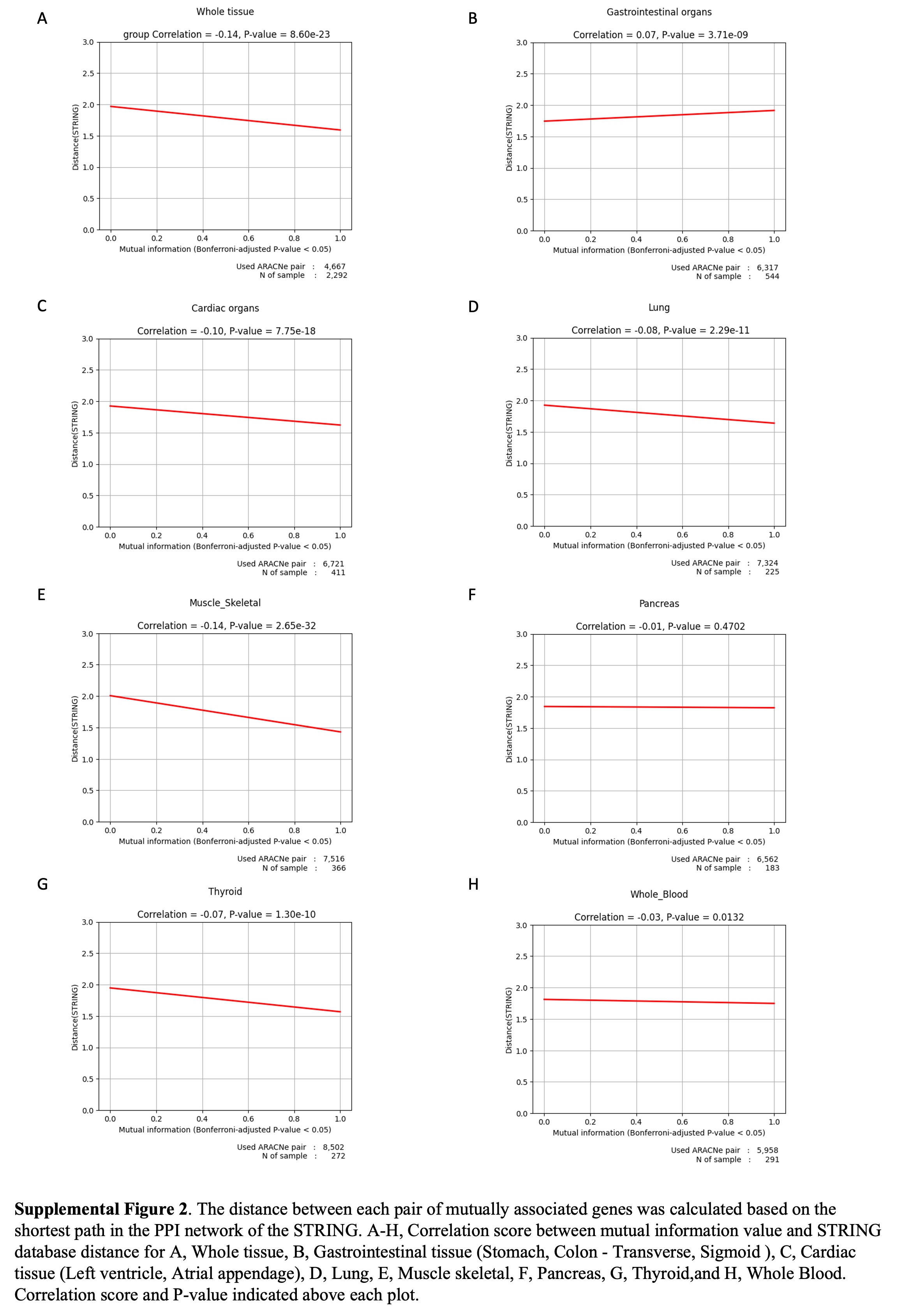

Supplement: baae111_Supp [file baae111_supp.zip › suppl_data/SFig2.jpg]
